# Antifibrotic effect of pirfenidone in a mouse model of human nonalcoholic steatohepatitis

**DOI:** 10.1038/srep44754

**Published:** 2017-03-17

**Authors:** Chikara Komiya, Miyako Tanaka, Kyoichiro Tsuchiya, Noriko Shimazu, Kentaro Mori, Shunsaku Furuke, Yasutaka Miyachi, Kumiko Shiba, Shinobu Yamaguchi, Kenji Ikeda, Kozue Ochi, Kazuhiko Nakabayashi, Ken-ichiro Hata, Michiko Itoh, Takayoshi Suganami, Yoshihiro Ogawa

**Affiliations:** 1 Department of Molecular Endocrinology and Metabolism, Graduate School of Medical and Dental Sciences, Tokyo Medical and Dental University, Tokyo, Japan; 2 Department of Molecular Medicine and Metabolism, Research Institute of Environmental Medicine, Nagoya University, Nagoya, Japan; 3 Department of Maternal-Fetal Biology, National Research Institute for Child Health and Development, Tokyo, Japan; 4 Department of Organ Network and Metabolism, Graduate School of Medical and Dental Sciences, Tokyo Medical and Dental University, Tokyo, Japan; 5 Department of Medical and Bioregulatory Science, Graduate School of Medical Sciences, Kyushu University, Fukuoka, Japan; 6 Japan Agency for Medical Research and Development, CREST, Tokyo, Japan

## Abstract

Non-alcoholic steatohepatitis (NASH) is characterized by steatosis with lobular inflammation and hepatocyte injury. Pirfenidone (PFD) is an orally bioavailable pyridone derivative that has been clinically used for the treatment of idiopathic pulmonary fibrosis. However, it remains unknown whether PFD improves liver fibrosis in a mouse model with human NASH-like phenotypes. In this study, we employed melanocortin 4 receptor-deficient (MC4R-KO) mice as a mouse model with human NASH-like phenotypes to elucidate the effect and action mechanisms of PFD on the development of NASH. PFD markedly attenuated liver fibrosis in western diet (WD)-fed MC4R-KO mice without affecting metabolic profiles or steatosis. PFD prevented liver injury and fibrosis associated with decreased apoptosis of liver cells in WD-fed MC4R-KO mice. Pretreatment of PFD inhibited the tumor necrosis factor-α (TNF-α)-induced liver injury and fibrogenic responses associated with decreased apoptosis of liver cells in wild-type mice. PFD also prevented TNF-α-induced hepatocyte apoptosis *in vitro* with reduced activation of caspase-8 and -3. This study provides evidence for the antifibrotic effect of PFD in a mouse model of human NASH. The data of this study highlight hepatocyte apoptosis as a potential therapeutic target, and suggest that PFD can be repositioned as an antifibrotic drug for human NASH.

Fibrosis is a pathological scarring process that leads to the destruction of organ architecture and impairment of organ function. Chronic dysfunction of parenchymal organs such as heart, intestine, kidney, liver, and lung is associated with fibrosis, thus accounting for an estimated one third of natural deaths worldwide^1^. Non-alcoholic fatty liver disease (NAFLD) is recognized as a hepatic phenotype of the metabolic syndrome. It encompasses a wide spectrum of liver impairment ranging from simple steatosis to non-alcoholic steatohepatitis (NASH), which is characterized by steatosis with lobular inflammation and fibrosis. NASH is currently recognized as a major cause of liver fibrosis in developed countries^2^,^3^, and it potentially progress to cirrhosis and hepatocellular carcinoma.

The pathogenesis of NASH is thought to be a multistep process in which steatosis is the initial step, and the macrophage-induced inflammatory response is important for the subsequent steps toward fibrosis^4^. During the development of NASH, hepatocytes store excessive lipid such as saturated fatty acid and free cholesterol, thereby leading to increased oxidative and endoplasmic reticulum stresses, which make parenchymal hepatocytes sensitive to apoptosis^5^. Apoptosis finally may be initiated by the Kupffer cell-derived tumor necrosis factor (TNF)-α and activated lymphocytes-derived FasL^5^,^6^. Apoptotic hepatocytes are thought to be engulfed by interstitial resident macrophages, which in turn lead to their activation. Some of the activated macrophages are reported to produce transforming growth factor (TGF)-β to secrete collagen from activated hepatic stellate cells (HSC), finally leading to fibrosis^5^. Although the multistep pathogenesis of NASH suggests its potential therapeutic targets according to the disease progression, there are currently no established pharmacological therapies for NASH.

Pirfenidone (PFD), an orally bioavailable pyridone derivative, has been clinically used for the treatment of idiopathic pulmonary fibrosis^7^,^8^. Although treatment with PFD for 24 months improved liver inflammation and fibrosis in patients with chronic hepatitis C^9^, the mechanism of its action is totally unknown. Moreover, whether PFD is effective in NASH has not been tested even in animal studies.

We have developed an experimental mouse model of NASH; melanocortin 4 receptor-deficient (MC4R-KO) mice fed a high fat diet or a western diet (WD) develop a liver condition similar to human NASH, being associated with obesity, insulin resistance and dyslipidemia^10^. Using this model, we have discovered the unique histological structure termed hepatic crown-like structures (hCLS) in the NASH-like liver, where dead or dying hepatocytes with large lipid droplets are surrounded by macrophages^11^. In hCLS, macrophages are considered to interact with dead parenchymal hepatocytes and fibrogenic cells, thereby accelerating inflammation and fibrosis in the liver.

Here we show that PFD markedly attenuates liver fibrosis in the rodent model of human NASH in parallel with significant reduction of hepatocyte apoptosis and hCLS formation. We also find that PFD inhibits the TNF-α-induced liver injury and fibrogenic responses *in vivo*, and indeed inhibits directly the TNF-α-induced hepatocyte apoptosis *in vitro*. This study provides evidence that PFD is capable of suppressing the progression of NASH through the inhibition of hepatocyte apoptosis. Collectively, we postulate that PFD may be repositioned as an antifibrotic therapeutic option against NASH.

## Results

### PFD prevents WD-fed MC4R-KO mice from liver fibrosis without affecting hepatic steatosis

**Figure 1:**
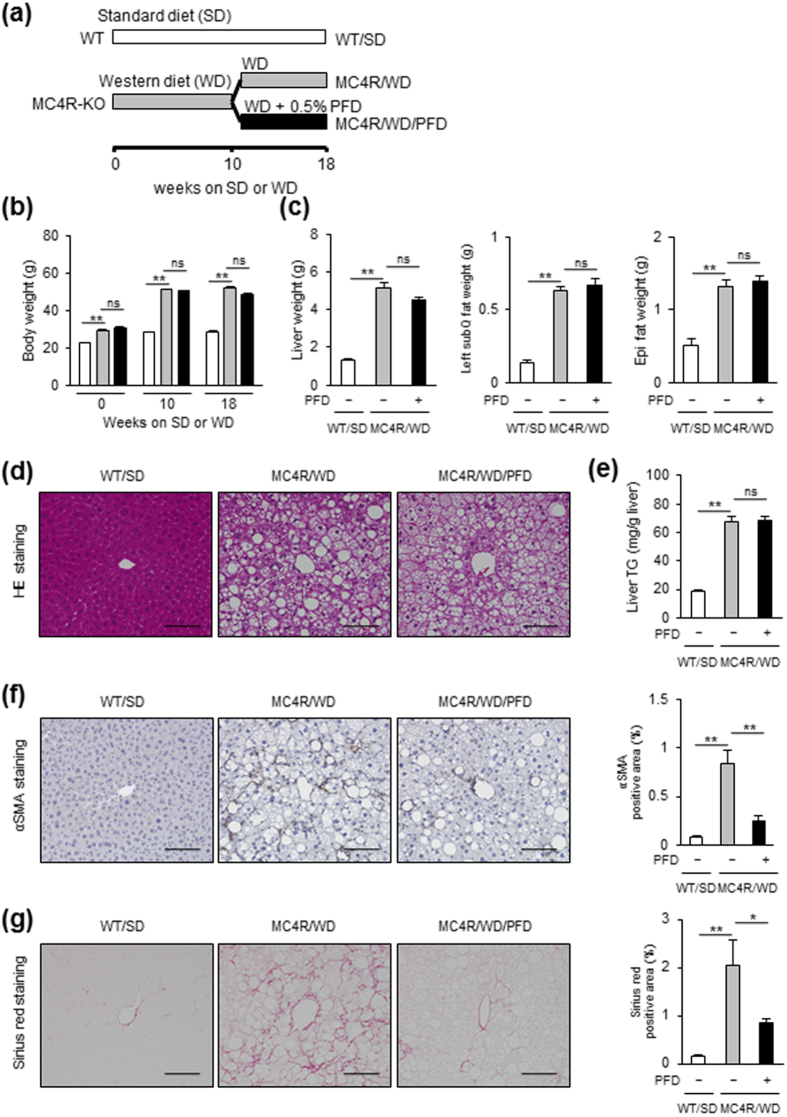
PFD prevents WD-fed MC4R-KO mice from liver fibrosis without affecting steatosis. (**a**) Study protocol. MC4R-deficient (MC4R-KO) mice were fed a Western diet (WD) for 10 weeks, and then fed a WD with or without PFD for 8 weeks. Control wild-type (WT) mice were fed a standard diet (SD) throughout the experiment period. (**b**) Body weight, and (**c**) weights of liver, and subcutaneous (subQ) and epididymal (Epi) fat tissue. (**d**) Hematoxylin and eosin (HE) staining, and (**e**) triglyceride (TG) content of the liver. Representative images and quantification of (**f**) α-SMA immunostaining and (**g**) Sirius red staining of the liver sections. Original magnification, ×200. Scale bars, 100 μm. *p < 0.05, **p < 0.01; ns, not significant. *n* = 6–8.

We examined whether PFD prevents the development of NASH in MC4R-KO mice during a WD feeding (Fig. 1a). The WD-fed MC4R-KO mice gained weight with increased liver and adipose tissue weight relative to standard diet (SD)-fed WT mice, which were unaffected by PFD (Fig. 1b and c). Metabolic analysis revealed that PFD does not affect systemic glucose and lipid metabolism in MC4R-KO mice (Table 1 and Supplementary Fig. S1). Moreover, PFD did not change lipid deposition or triglyceride (TG) accumulation in the liver of WD-fed MC4R-KO mice (Fig. 1d and e). In contrast, PFD significantly attenuated the otherwise elevated serum alanine aminotransferase (ALT) levels in WD-fed MC4R-KO mice (Table 1). Furthermore, PFD inhibited HSC activation in WD-fed MC4R-KO mice, as assessed by immunohistochemistry for α-SMA (Fig. 1f). These observations suggest that PFD prevents MC4R-KO mice from liver injury independent of hepatic steatosis during a WD feeding. Accordingly, PFD significantly reduced liver fibrosis in WD-fed MC4R-KO mice (Fig. 1g).

**Figure 2:**
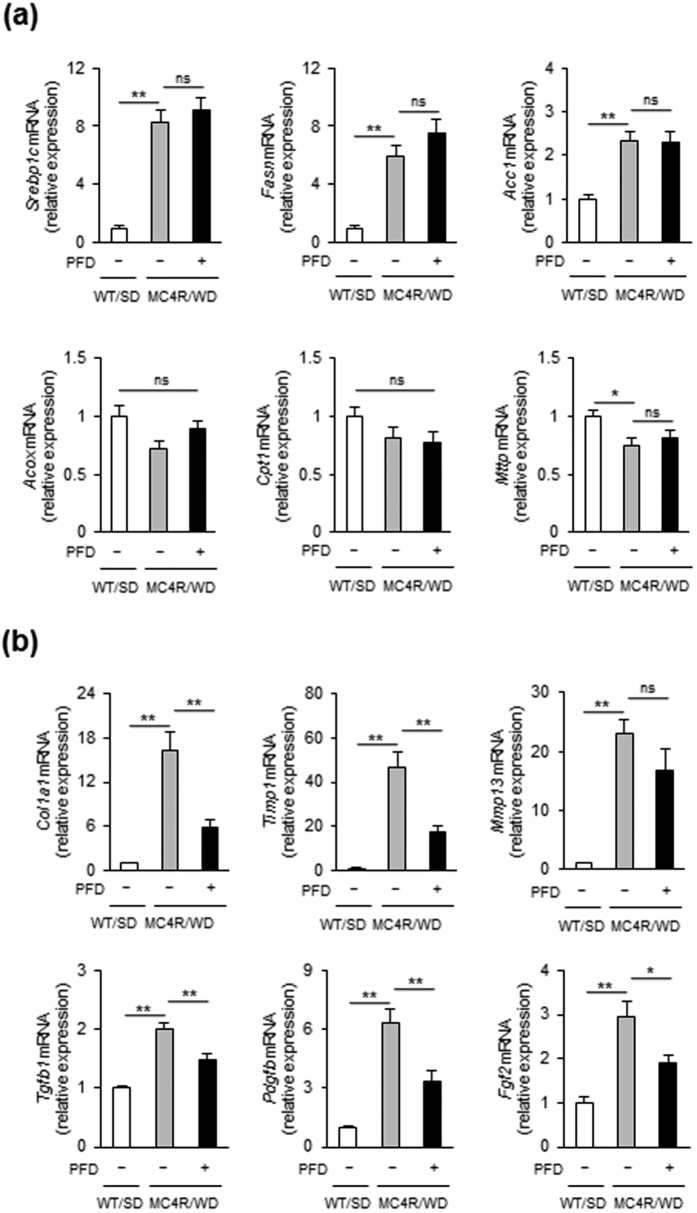
PFD attenuates upregulation of fibrosis-related genes in the liver of WD-fed MC4R-KO mice. mRNA expression of genes related to (**a**) lipid metabolism and (**b**) fibrosis in the liver. *p < 0.05, **p < 0.01; ns, not significant. *n* = 6–8.

In the liver from WD-fed MC4R-KO mice, PFD did not affect expression of *de novo* lipogenic genes such as sterol regulatory element-binding protein-1c (*Srebp1c*), fatty acid synthase (*Fasn*), and acetyl-CoA carboxylase (*Acc1*) as well as fatty acid β-oxidation genes such as acyl-CoA oxidase 1 (*Acox1*) and carnitine palmitoyltransferase 1a, liver (*Cpt1a*), and triglyceride transfer protein (*Mttp*) (Fig. 2a). In contrast, PFD dramatically suppressed the induction of collagen type I α1 gene (*Col1a1*) in the liver of WD-fed MC4R-KO mice (Fig. 2b). Induction of *Tgfb1, Pdgfb* and *Fgf2*, all of which are key cytokines of activation and proliferation of HSC, were also inhibited by PFD (Fig. 2b).

### PFD reduces hepatic inflammation and hCLS formation in WD-fed MC4R-KO mice

We histologically assessed the severity of liver injury with the NAFLD Activity Score (NAS); the WD-fed MC4R-KO mice showed a significantly greater level of steatosis, lobular inflammation, and hepatocyte ballooning than WT mice fed an SD, thus resulting in a higher overall NAS score (Table 2). Whereas PFD unchanged the steatosis score, the scores for lobular inflammation and hepatocyte ballooning were decreased by PFD. As a result, PFD lowered NAS in WD-fed MC4R-KO mice (Table 2).

**Figure 3:**
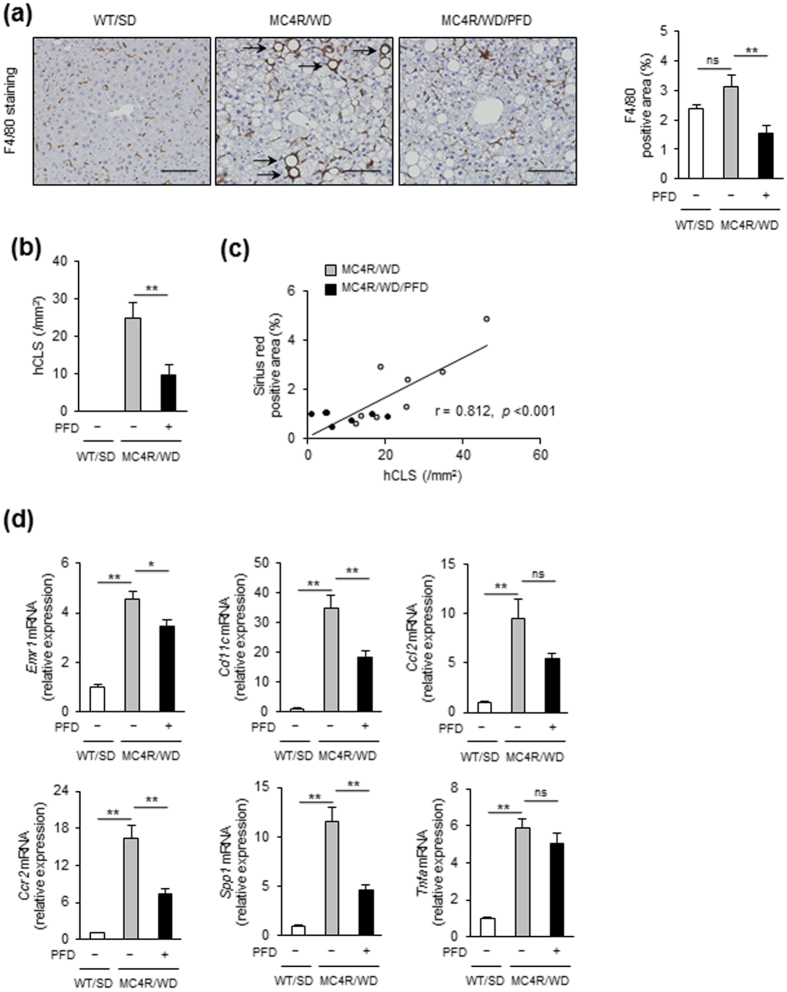
PFD inhibits hCLS formation in the liver of WD-fed MC4R-KO mice. (**a**) Representative images and quantification of F4/80 immunostaining of the liver sections from WD-fed MC4R-KO mice after treatment with PFD. Arrows indicate “hepatic crown-like structure (hCLS)”. (**b**) Quantification of hCLS number in the liver sections. (**c**) Correlation of the number of hCLS with the Sirius red-positive area. (**d**) mRNA expression of genes related to macrophage markers, proinflammatory cytokines and chemokines in the liver. Original magnification, ×200. Scale bars, 100 μm. *p < 0.05, **p < 0.01; ns, not significant. *n* = 6–8.

We previously reported a unique histological structure or hCLS in the liver from MC4R-KO mice, where dead hepatocytes are surrounded by macrophages and suggested that hCLS promotes liver fibrosis during the progression from simple steatosis to NASH^11^. Immunostaining for a macrophage marker F4/80 revealed that PFD treatment effectively suppresses F4/80-positive area and hCLS formation in MC4R-KO mice (Fig. 3a and b). The number of hCLS was positively correlated with fibrosis area as we previously reported^11^ (Fig. 3c).

In accordance with decreased hCLS formation, PFD reduced expression of *Emr1* and *Cd11c* in the liver of WD-fed MC4R-KO mice (Fig. 3d). Expression of C-C chemokine ligand 2 (*Ccl2*), which promotes macrophage recruitment^12^,^13^, trended to be reduced in PFD-treated WD-fed MC4R-KO mice. Moreover, expression of C-C chemokine receptor 2 (*Ccr2*), a receptor of C-C chemokine ligand 2, and osteopontin (*Spp1*) were significantly reduced by PFD. Expression of TNF-α (*Tnfa*), which has been reported to accelerate apoptosis of hepatocytes in a murine model of NAFLD^5^,^14^, was higher in WD-fed MC4R-KO mice than that in SD-fed WT mice. In this study, PFD did not affect *Tnfa* expression in WD-fed MC4R-KO mice.

### PFD reduces cell death in the liver of MC4R-KO mice

**Figure 4:**
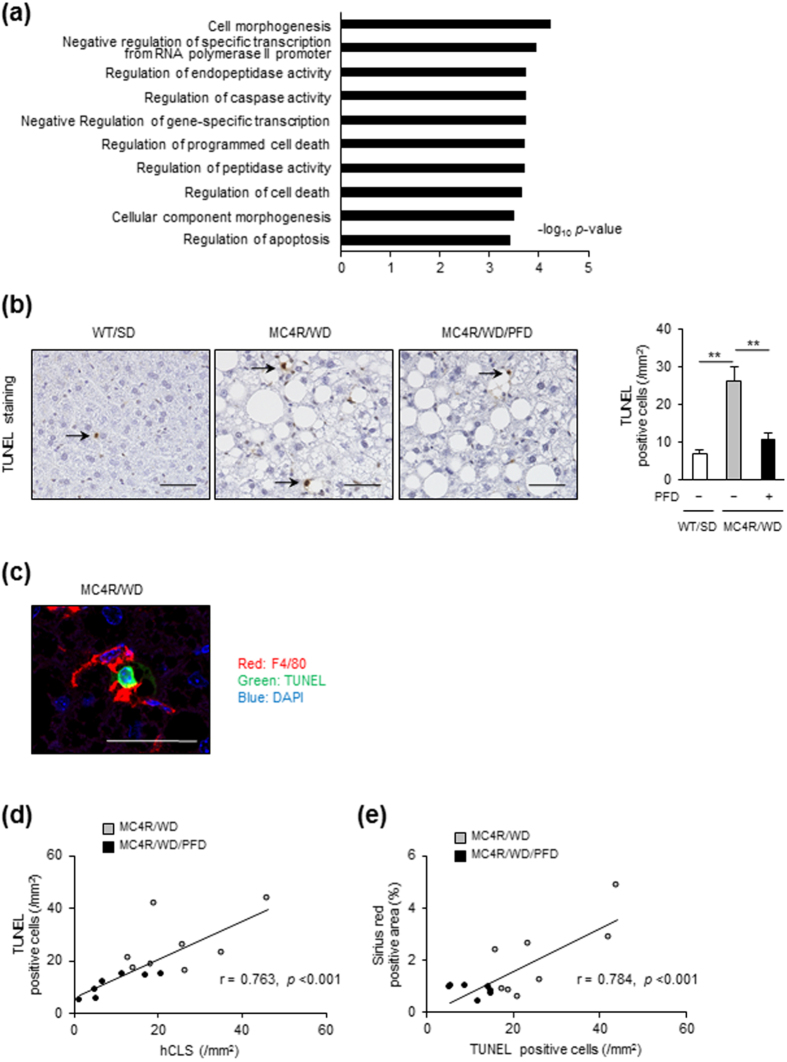
PFD reduces cell death in the liver of MC4R-KO mice. (**a**) The pathways enriched among the upregulated (>1.5-fold) mRNAs in the liver of WD-fed MC4R-KO mice treated with PFD for 8 weeks compared to those of WD-fed MC4R-KO mice without PFD treatment. The results are expressed as −log (p value). (**b**) Representative images and quantification of TUNEL-positive cells in the liver sections from MC4R-KO mice after treatment with PFD. (**c**) A representative image of F4/80-positive (red) cells surrounding a TUNEL-positive (green) cell. Blue: DAPI. (**d**) Correlation of the number of hCLS with that of TUNEL-positive cells. (**e**) Correlation of the number of TUNEL-positive cells with the Sirius red-positive area. Original magnification, ×200. Scale bars, 100 μm. *p < 0.05, **p < 0.01. *n* = 6–8.

To elucidate the molecular mechanisms by which PFD prevents hepatic inflammation and fibrosis in MC4R-KO mice, we performed DNA microarray analysis of the liver from WD-fed MC4R-KO mice treated with or without PFD. Gene ontology analysis revealed that genes related to cell cycle are enriched as the differentially expressed genes in the liver from WD-fed MC4R-KO mice treated with PFD, relative to untreated mice (Fig. 4a). In a 3-day treatment, genes related to cell death or apoptosis were enriched in the liver from WD-fed MC4R-KO mice treated with PFD (Supplementary Fig. S2).

Based on the data of microarray analysis, we next examined whether apoptosis in the liver is suppressed by PFD. Terminal deoxynucleotidyl transferase-mediated dUTP nick end labeling (TUNEL) staining revealed that the number of apoptotic cells is increased in WD-fed MC4R-KO mice relative to SD-fed WT mice, which is significantly reduced by PFD (Fig. 4b). Immunofluorescence staining demonstrated that there are TUNEL-positive cells surrounded by F4/80-positive macrophages (Fig. 4c). In this study, the number of TUNEL-positive cells was positively correlated with that of hCLS and fibrosis area (Fig. 4d and e).

### PFD attenuates TNF-α-induced liver injury in WT mice

**Figure 5:**
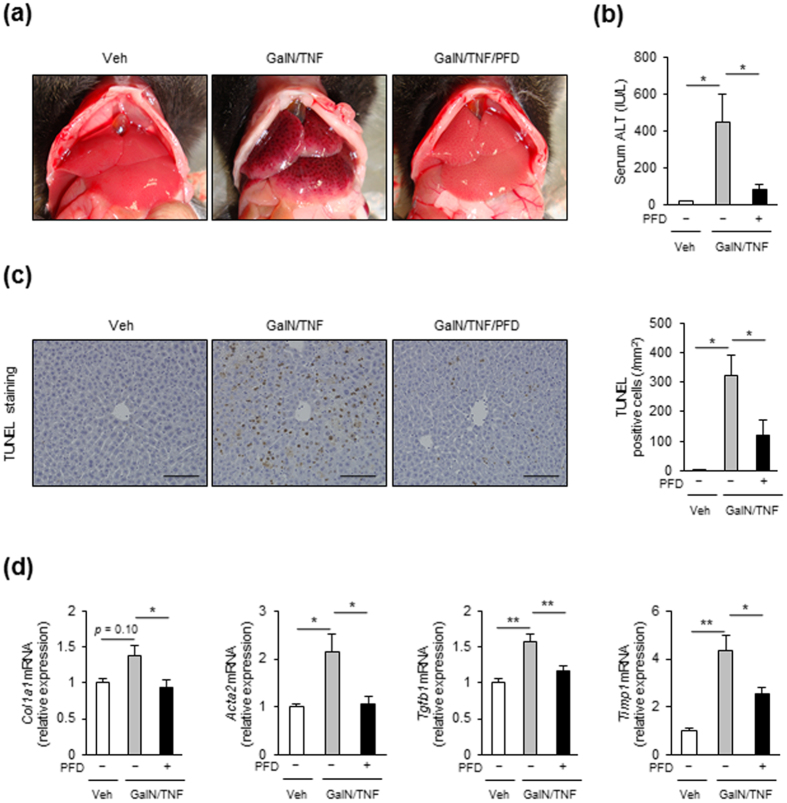
PFD attenuates TNF-α-induced liver injury in WT mice. (**a**) Representative images of the liver and (**b**) serum ALT levels in WT mice pretreated with or without PFD followed by vehicle (Veh), or D-galactosamine and TNF-α (GalN/TNF) administration. Mice were sacrificed after 5 h of GalN/TNF administration. (**c**) Representative images and quantification of TUNEL-positive cells in the liver sections. (**d**) mRNA expression of genes related to fibrosis. Original magnification, ×200. Scale bars, 100 μm. *p < 0.05, **p < 0.01. *n* = 6–8.

The reduced apoptotic cells in the liver of PFD-treated MC4R-KO mice allowed us to investigate the effect of PFD on hepatocyte apoptosis, which could be a primary mechanism of its anti-inflammatory and antifibrotic action. Indeed, upon PFD treatment, gene expression of death receptors (*Tnfrsf1a* and *Fas*) and their cognate ligands (*Tnfa* and *Fasl*) were consistently upregulated in the liver from MC4R-KO mice (Supplementary Fig. S3). In this study, we also examined the effects of PFD on TNF-α-induced liver injury, where GalN and TNF-α were concomitantly administered to WT mice to induce hemorrhagic hepatitis following hepatocyte apoptosis^15^. PFD dramatically attenuated gross liver hemorrhage and elevation of serum ALT in GalN/TNF-α-treated WT mice (Fig. 5a and b). TUNEL staining demonstrated that PFD markedly inhibits the GalN/TNF-α-induced hepatocyte apoptosis (Fig. 5c). The GalN/TNF-α-induced expression of fibrogenic genes such as *Col1a1, Acta2, Tgfb1*, and *Timp1* in WT mice were significantly blunted by PFD (Fig. 5d). These observations suggest that PFD inhibits the TNF-α-induced hepatocyte apoptosis and upregulation of fibrogenic genes in the liver of WT mice.

### PFD prevents TNF-α-induced hepatocyte apoptosis with reduced caspases-8 and -3 activities

**Figure 6:**
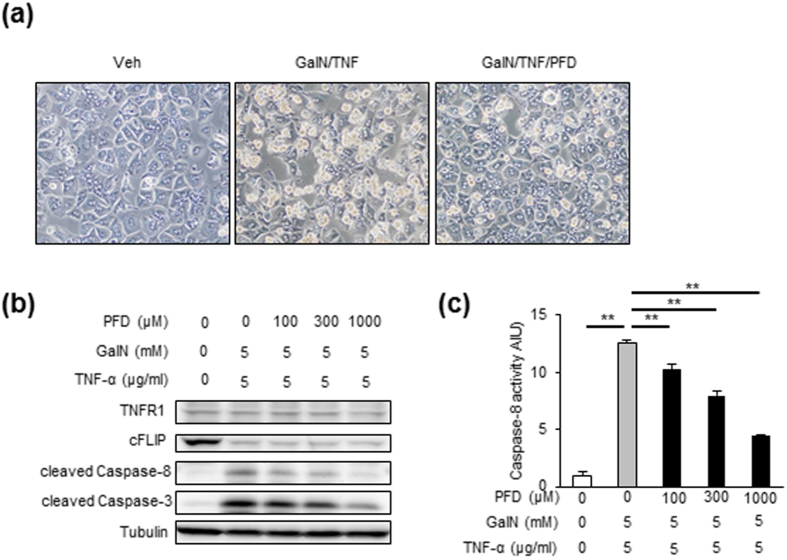
PFD inhibits TNF-α-induced apoptosis in primary hepatocytes with reduced caspase-8 and -3 activities. (**a**) Representative images of the primary hepatocytes from WT mice pretreated with or without PFD (1000 μM) followed by vehicle (Veh), or D-galactosamine and TNF-α (GalN/TNF) treatment for 16 h. (**b**) Representative western blots and (**c**) caspase-8 activities of primary hepatocytes pretreated with or without indicated concentrations of PFD followed by GalN/TNF treatment for 16 h. TNF-R1: tumor necrosis factor receptor 1, cFLIP: cellular FADD-like IL-1β-converting enzyme-inhibitory protein. **p < 0.01. *n* = 6–8.

We conducted *in vitro* experiments using primary hepatocytes to examine whether PFD directly inhibits hepatocyte apoptosis, and if so, its molecular mechanism. In response to GalN/TNF-α, primary hepatocytes were induced to cell death and detached from culture plates, which was markedly suppressed by PFD (Fig. 6a). Western blotting and caspase activity assay showed that PFD dose-dependently inhibits the GalN/TNF-α-induced caspases-8 and -3 activation in primary hepatocytes without affecting TNF-R1 expression (Fig. 6b and c). GalN/TNF-Αalso activated cIAP and JNK, and reduced an anti-apoptotic molecule cellular FADD-like IL-1β-converting enzyme-inhibitory protein (c-FLIP), which were not affected by PFD (Supplementary Fig. S4). Whereas PFD also inhibited another apoptotic signal Fas-mediated caspase-8 activation in primary hepatocytes, it did not attenuate caspase-3 activation (Supplementary Fig. S5a and S5b). PFD did not inhibit palmitate-induced caspase-8 and -3 activation in primary hepatocytes (Supplementary Fig. S5c).

In this study, PFD did not inhibit TGF-β-induced *Tgfb1* and *Col1a1* expression in HSC line LX-2 (Supplementary Fig. S6a). Moreover, PFD did not change the lipopolysaccharides (LPS)-induced *Tnfa, Ccl2, Tgfb1*, and *Timp1* expression in macrophage cell line RAW264.7 cells (Supplementary Fig. S6b).

## Discussion

Although PFD has ameliorated pharmacologically- and surgically-induced rodent models of liver injury and fibrosis^16^,^17^,^18^,^19^, whether PFD can be effective in a rodent model that closely reflects the liver condition of human NASH has not been addressed so far. Here we demonstrate that PFD prevents the development of hepatic inflammation and fibrosis in the liver from WD-fed MC4R-KO mice; a recently developed rodent model of human NASH^10^. This study has our findings with the MC4R-KO mice have a significant clinical impact on the treatment of human NASH with PFD.

It is noteworthy that PFD prevents liver injury and fibrosis without affecting metabolic profiles and steatosis. These observations suggest that PFD inhibits the progression of NASH at least after hepatic steatosis. According to the original “two-hit” hypothesis, the pathogenesis of NASH may involve at least two distinct processes; excessive accumulation of lipids in the liver and enhanced liver injury and fibrosis^20^. More recently, the “multiple parallel hit” hypothesis has been proposed, which comprises multiple hits acting in parallel during the progression of simple steatosis to NASH^4^. These observations suggest that the NASH pathogenesis can be roughly dissociated into two processes; hepatic steatosis caused by metabolic abnormalities, and following hepatic inflammation toward fibrosis.

In this study, PFD decreased the number of TUNEL-positive cells and hCLS in the liver of WD-fed MC4R-KO mice. Because hCLS serves as an origin of hepatic inflammation and fibrosis, it is likely that PFD primarily inhibits hepatocyte apoptosis, thereby preventing the fibrogenic response in the liver of WD-fed MC4R-KO mice. Apoptosis has been recognized as a key player in the initiation and propagation of nearly all types of organ fibrosis^21^. Enhanced apoptosis is critical for the development of organ fibrosis, and multiple mechanisms by which the apoptotic cells might dictate fibrotic outcomes have been suggested^22^; apoptotic cells can stimulate macrophages, neutrophils, and other leukocytes to secrete factors that mediate fibrotic effects following the engulfment of apoptotic cells in various organs^23^. Thus, apoptosis could be a fundamental biological process with wide-ranging implications in fibrosis, and thus, a potential therapeutic target. It is noteworthy that PFD is antifibrotic in various animal models of fibrosis in the lung^24^,^25^,^26^, heart^27^,^28^, and kidney^29^,^30^. It is likely that the antiapoptic effect of PFD is involved in the prevention for fibrosis in the various organs as a common mechanism.

Recent studies have demonstrated that hepatocyte apoptosis is increased in patients with NASH, which is correlated with the disease severity and stage of fibrosis in the liver^5^,^31^,^32^. In this study, we found that gene expression of death receptors; *Tnfrsf1a* and *Fas*, and their cognate ligands; *Tnfa* and *Fasl*, are upregulated in the liver from WD-fed MC4R-KO mice. This is consistent with a couple of previous reports on human NASH^32^,^33^. Importantly, PFD treatment only for 3 days changed genes related to cell death or apoptosis in the liver. It is, therefore, likely that the antifibrotic effect of PFD in the liver from WD-fed MC4R-KO mice involves its direct effect on cell death or apoptosis. This discussion is supported by our observation that PFD inhibits potently the TNF-α-induced hepatocyte apoptosis and liver injury in WT mice.

PFD also inhibited the TNF-α- and Fas ligation-induced caspase-8 activation in primary hepatocytes. These observations suggest that PFD prevents the TNF-α-induced hepatocyte apoptosis *via* inhibiting caspase-8 activation. There is a previous report that hepatocyte-specific ablation of caspase-8 markedly attenuates methionine choline deficient diet-induced liver injury and fibrosis in mice, which is associated with reduced number of apoptotic cells and hepatic inflammation^34^. Moreover, some pan-caspase inhibitors are reported to prevent liver fibrosis in experimental animal models^35^,^36^,^37^, and to reduce aminotransferase levels in patients with NASH^38^,^39^,^40^. Unlike TNF-α-induced hepatocyte apoptosis, *in vitro* experiments have shown that PFD attenuated Fas ligation-induced activation of caspase-8, but not caspase-3. In addition, PFD did not inhibit palmitate-induced caspase activation in primary hepatocytes. It therefore suggests that effect of PFD on death receptor signaling is a pathway- and receptor-dependent; PFD may have little effect on a pathway of caspase-8-independent caspase-3 activation by Fas ligation^41^,^42^, and TNF-related apoptosis-inducing ligand receptor 2 (TRAIL-R2)-mediated caspase activation by palmitate stimulation^43^. Although the detailed molecular mechanisms by which PFD inhibits apoptosis and caspase activation in hepatocytes remain to be elucidated, it is conceivable that inhibition of hepatocyte caspase-8 activation, possibly triggered by TNF-α, appears to be a primary mechanism of PFD action to prevent WD-fed MC4R-KO mice from liver injury and fibrosis.

Although many basic and clinical studies have been carried out to develop therapeutic strategies against NASH, there are currently few drugs with proven efficacy^44^,^45^. Indeed, clinical evidence is limited regarding their long-term safety. Whereas gastrointestinal and skin-related adverse events have been reported, PFD has relatively a favorable safety profile for the treatment of idiopathic pulmonary fibrosis in clinical trials, observational studies, and real-world use^7^,^8^. Together with the beneficial effect of PFD treatment improves hepatic inflammation and fibrosis in patients with chronic hepatitis C^9^, the data of this study suggest that PFD can be repositioned as an antifibrotic drug in treatment of human NASH.

In conclusion, this study provides evidence that PFD exerts its antifibrotic effect at least in part through the inhibition of cell death in a mouse model of human NASH. Given its safety profile in idiopathic pulmonary fibrosis, we postulate that PFD may be repositioned as an antifibrotic drug in NASH. The data of this study also unravel an antifibrotic mechanism of PFD, and the pathophysiological role of hepatocyte apoptosis in the development of NASH, and thus suggest a clinical implication for the treatment of NASH.

## Mathods

### Ethics statement

This study was carried out in accordance with the guidelines for the care and use of laboratory animals of Tokyo Medical and Dental University. The protocol was approved by Tokyo Medical and Dental University Committee on Animal Research (2011-207C3, 0140016 A).

### Animals and experimental protocol

The MC4R-KO mice on the C57BL/6 J background were a gift from Dr. Joel K. Elmquist (University of Texas Southwestern Medical Center, Dallas, TX, USA)^46^. Male C57BL/6 J WT mice were purchased from CLEA Japan, Inc. (Tokyo, Japan). The animals were allowed free access to water and a SD (CE-2; 343 kcal/100 g, 12.6% energy as fat; CLEA Japan Inc.). Eight-week-old male MC4R-KO mice were fed a WD (D12079B; 468 kcal/100 g, 41% energy as fat, 34% sucrose, 0.21% cholesterol; Research Diets Inc., New Brunswick, NJ, USA) for 10 weeks, and thereafter fed a WD with or without PFD (provided by Shionogi & Co., Ltd., Osaka, Japan) for 8 weeks (Fig. 1a). PFD was added to a WD at 0.5%, when mice treated took approximately 280 mg/kg/day of PFD. Eight-week-old control male WT mice were fed a SD throughout the experiment period. At the end of the experiment, the animals were sacrificed under anesthesia after 1 h of fasting.

### Glucose and insulin tolerance tests

For glucose tolerance tests, mice were fasted for 16 h with free access to water followed by intraperitoneal glucose injection (1.5 g/kg). For insulin tolerance tests, after 2 h of fasting, mice were intraperitoneally injected with human insulin (1.0 U/kg). We measured blood glucose levels at 0, 30, 60, 90, and 120 min after the glucose or insulin injection.

### TNF-α-induced liver injury

Eight-week-old male WT mice were pretreated with 500 mg/kg of PFD suspended in 0.5% Methyl Cellulose 400 Solution (MC, Wako Pure Chemical Industries, Ltd., Osaka, Japan) or 0.5% MC by gavage. After 15 min, mice were intraperitoneally injected with 700 mg/kg of D-galactosamine (GalN, Nacalai Tesque Inc., Kyoto, Japan) dissolved in phosphate buffer saline (PBS). After 1 h of GalN injection, 20 μg/kg of recombinant mouse TNF-α (R&D Systems, Inc., Minneapolis, MN, USA) dissolved in PBS with 0.1% bovine serum albumin (BSA, Sigma-Aldrich Co., St. Louis, MO, USA) were intraperitoneally injected. Control mice were given 0.5% MC by gavage, and then received intraperitoneal injection of PBS and 0.1% BSA as vehicle. The animals were sacrificed under anesthesia at 5 h after TNF-α injection.

### Biochemical assays

Blood glucose was measured using a glucometer (Glutest PRO R; Sanwa Kagaku Kenkyusho Co., Ltd., Aichi, Japan). Serum insulin level was measured with an enzyme-linked immunosorbent assay kit (Morinaga Institute of Biological Science, Inc., Kanagawa, Japan). Serum ALT, non-esterified fatty acid (NEFA), triglyceride (TG), and total cholesterol (TC) levels were analyzed with enzymatic assays in a laboratory of Ikagaku Co., Ltd. (Kyoto, Japan). Total lipids were extracted from the liver with chloroform and methanol (2:1 v/v), and liver TG content was assayed with TG E-Test Wako (Wako Pure Chemical Industries, Ltd.).

### Histological analysis

The liver was fixed with neutral-buffered formalin and embedded in paraffin. Liver sections were stained with hematoxylin and eosin (HE) and Sirius red. Activated HSC and macrophages were detected immunohistochemically using a α-smooth muscle actin (α-SMA) antibody (ab5694, Abcam plc, Cambridge, UK) and a rat monoclonal F4/80 antibody^47^. TUNEL staining was performed using the ApopTag Plus Peroxidase *In Situ* Apoptosis Detection Kit (Millipore, Billerica, MA, USA) according to the manufacturer’s instruction. NAFLD activity score (NAS) was determined according to the published criteria^48^. The Sirius red, α-SMA, and F4/80-positive areas were measured using an image analysis software (WinROOF; Mitani Corporation, Tokyo, Japan). Hepatic crown-like structures (hCLS) and TUNEL positive cells were counted in the whole area of the section. Histological scoring and quantification were performed by 2 investigators.

### Cell culture

LX-2 cells were kindly provided from Dr. Scott L. Friedman (Mount Sinai School of Medicine, New York, NY, USA). RAW264.7 cells were purchased from RIKEN BioResource Center (Ibaraki, Japan). Primary hepatocytes were isolated and cultured as previously described^49^. Eight-week-old male WT mice were anesthetized, and the liver was perfused via the portal vein with Hank’s Balanced Salt Solution (HBSS, Nacalai tesque Inc.) containing 0.5 mM ethylene glycol tetraacetic acid (EGTA, Nacalai tesque Inc.), followed by Dulbecco’s Modified Eagle Medium (DMEM, Nacalai tesque Inc.) containing 75 mg/dl collagenase type IV (Worthington Biochemical Corp., Lakewood, NJ, USA). The liver was suspended in DMEM supplemented 10% fetal bovine serum (FBS, Biowest, Nuaille, France), and primary hepatocytes were purified using density gradient centrifugation (45% Percoll, Sigma-Aldrich Co.). Cells were cultured in a humidified incubator at 37 °C and 5% CO_2_.

### TNF-α and palmitate treatment, and Fas ligation in primary hepatocytes

Primary hepatocytes were plated in DMEM supplemented with 10% FBS in 6-well plates. After 4 h of incubation, the medium was replaced with DMEM without FBS. For stimulation with TNF-α, primary hepatocytes were pretreated with or without PFD (0–1000 μM) for 4 h, and then treated with 5 mM of GalN and 5 ng/ml of recombinant mouse TNF-α for 12–16 h. For stimulation with palmitate, primary hepatocytes were pretreated with or without PFD (0–1000 μM) for 4 h, and then treated with 250 μM of palmitate for 24 h. For Fas ligation, primary hepatocytes were pretreated with or without PFD (0–1000 μM) for 4 h, thereafter treated with 0.5 μg/ml anti-mouse Fas/CD95 antibody (Jo2, BD Biosciences, San Jose, CA, USA) for 6 h.

### Caspase-8 assay

Caspase-8 activity was measured using APOPCYTO caspase-8 Fluorometric Assay Kit (Medical & Biological Laboratories Co., Ltd., Aichi, Japan) according to the manufacturer’s instructions. Relative activity was calculated as the ratio of the luminescence obtained from treated/untreated samples and standardized to protein concentrations.

### Western blotting

Primary hepatocytes were lysed in a lysis buffer (2% SDS, 4 M Urea, 1 mM EDTA, 150 mM NaCl, 50 mM Tris pH 8.0) supplemented with Halt Protease and Phosphatase Inhibitor Cocktail (Thermo Fisher Scientific Inc., Waltham, MA, USA). Immunoblotting was performed with a TNF-R1 (sc-8436, Santa Cruz Biotechnology, Santa Cruz, CA, USA), Cleaved Caspase-8 (Asp387) (8592, Cell Signaling Technology, Danvers, MA, USA), Caspase-3 (ab13847, Abcam plc), c-IAP1 (4952, Cell Signaling Technology), c-IAP2 (sc-7944. Santa Cruz Biotechnology), c-FLIP (Dave-2, Adipogen Corporation, San Diego, CA, USA), p-JNK (sc-6254, Santa Cruz Biotechnology), JNK (9252, Cell Signaling Technology), and α-Tubulin antibody (2144, Cell Signaling Technology). Immunoblots were detected and analyzed with ECL Prime Western Blotting Detection Reagent and ImageQuant LAS 4000 mini (GE Healthcare, Little Chalfont, UK).

### Quantitative RT-PCR

Total RNA of the liver was isolated using Sepasol reagent (Nacalai Tesque Inc.). RNA was reverse transcribed with Random Primer (Thermo Fisher Scientific Inc.) and ReverTra Ace (Toyobo Co., Ltd., Osaka, Japan). Quantitative RT-PCR was performed using StepOnePlus Real-time PCR System with Fast SYBR Green Master Mix Reagent (Thermo Fisher Scientific Inc.). Primer sequences are available upon request. Data were normalized to the *36b4* levels, and analyzed by the comparative CT method.

### Microarray analysis

Total RNA of the liver was isolated using RNeasy Plus Universal Mini Kit (Qiagen, Hilden, Germany). Microarray analysis was performed using Agilent SurePrint G3 Mouse GE 8 × 60 K (Agilent Technologies, Inc., Santa Clara, CA, USA) in a laboratory of DNA Chip Research Inc. (Tokyo, Japan). Differentially expressed genes were determined by fold change (>1.5), and gene ontology analysis was performed using DAVID Bioinformatics Resources 6.7.

### Statistical analysis

Data are expressed as mean ± standard error of the mean (SEM). Data were compared using analysis of variance (ANOVA) with post hoc testing. Pearson correlation coefficient was used to evaluate correlations between variables. *p* < 0.05 was considered to be statistically significant. Statistical analysis was performed using Prism 6 (GraphPad software, Inc., La Jolla, CA, USA).

## Additional Information

**How to cite this article**: Komiya, C. *et al*. Antifibrotic effect of pirfenidone in a mouse model of human nonalcoholic steatohepatitis. *Sci. Rep.* **7**, 44754; doi: 10.1038/srep44754 (2017).

**Publisher's note:** Springer Nature remains neutral with regard to jurisdictional claims in published maps and institutional affiliations.

## Supplementary Material

Supplementary Information

## Figures and Tables

**Table 1:** Effect of PFD treatment on serum parameters in NASH.

	WT/SD	WD/MC4R-KO	WD/MC4R-KO/PFD
BG (mg/dl)	129.7 ± 6.6	145.3 ± 5.4	170.4 ± 9.2^a,b^
Insulin (ng/ml)	0.88 ± 0.47	5.96 ± 1.50	7.25 ± 1.65^a^
TC (mg/dl)	69.5 ± 1.8	279.4 ± 26.2^a^	219.4 ± 13.3^a^
TG (mg/dl)	33.0 ± 0.7	34.9 ± 2.9	30.9 ± 3.6
FFA (mEq/l)	636.7 ± 57.0	707.5 ± 20.2	607.1 ± 46.1
ALT (IU/l)	20.0 ± 1.2	403.9 ± 1.2^a^	268.5 ± 40.0^b^

WT, wildtype; SD, standard diet; WD, western diet; BG, blood glucose; TC, total cholesterol; TG, triglyceride; FFA, free fatty acid; ALT, alanine aminotransferase. Data are mean ± SEM. ^a^*p* < 0.05 vs. WT-SD; ^b^p < 0.05 vs. MC4R-KO-WD. n = 6–8.

**Table 2:** Effect of PFD treatment on liver histology in NASH.

		WD/MC4R-KO	WD/MC4R-KO/PFD
Steatosis	0.0 ± 0.0	3.0 ± 0.0^a^	3.0 ± 0.0^a^
Inflammation	0.0 ± 0.0	2.3 ± 0.3^a^	1.1 ± 0.3^a,b^
Ballooning	0.0 ± 0.0	1.9 ± 0.1^a^	1.0 ± 0.3^a,b^
NAS	0.0 ± 0.0	7.1 ± 0.4^a^	5.1 ± 0.6^a,b^

WT, wildtype; SD, standard diet; WD, western diet. Data are mean ± SEM. ^a^*p* < 0.05 vs. WT-SD; ^b^p < 0.05 vs. MC4R-KO-WD. n = 6–8.
