# Supplementary material for: Antifibrotic effect of pirfenidone in a mouse model of human nonalcoholic steatohepatitis
Source: Sci Rep. 2017 Mar 17;7:44754. doi: 10.1038/srep44754 (PMC5355985; doi:10.1038/srep44754)
Supplement: Supplementary Information [file srep44754-s1.pdf]

Antifibrotic effect of pirfenidone in a mouse model of human nonalcoholic steatohepatitis

Chikara Komiya[1,7], Miyako Tanaka[2,7], Kyoichiro Tsuchiya[1]*, Noriko Shimazu[1], Kentaro Mori[1], Shunsaku Furuke[1], Yasutaka Miyachi[1], Kumiko Shiba[1], Shinobu Yamaguchi[1], Kenji Ikeda[1], Kozue Ochi[2], Kazuhiko Nakabayashi[3], Ken-ichiro Hata[3], Michiko Itoh[1,4], Takayoshi Suganami[2], and Yoshihiro Ogawa[1,5,6]*

[1]Department of Molecular Endocrinology and Metabolism, [4]Department of Organ Network and Metabolism, Graduate School of Medical and Dental Sciences, Tokyo Medical and Dental University, Tokyo, Japan; [2]Department of Molecular Medicine and Metabolism, Research Institute of Environmental Medicine, Nagoya University, Nagoya, Japan; [3]Department of Maternal-Fetal Biology, National Research Institute for Child Health and Development, Tokyo, Japan; [5]Department of Medical and Bioregulatory Science, Graduate School of Medical Sciences, Kyushu University; [6]Japan Agency for Medical Research and Development, CREST, Tokyo, Japan.

[7]These authors contributed equally to this work.

Supplementary Figure Legends

Supplementary Fig. S1. PFD does not affect glucose tolerance and insulin sensitivity in MC4R-KO mice.

(a) Glucose and (b) insulin tolerance tests for SD-fed WT mice and WD-fed MC4R-KO with or without PFD treatment for 8 weeks.

Supplementary Fig. S2. Short-term treatment of PFD changes cell cycle-related genes in the liver of MC4R-KO mice.

The pathways enriched among the upregulated (> 1.5-fold) mRNAs in the liver of WD-fed MC4R-KO mice treated with PFD for 3 days compared to those of WD-fed MC4R-KO mice without PFD treatment. The results are expressed as -log (*p* value).

Supplementary Fig. S3. Ten-week feeding of WD upregulates genes of death receptors and their respective ligands in the liver of MC4R-KO mice.

mRNA expression of genes related to death receptors and their respective ligands in the liver. * $p < 0.05$, ** $p < 0.01$. $n = 6\text{-}8$.

Supplementary Fig. S4. PFD does not affect TNF-α-induced IAP and JNK1/2 activation in primary hepatocytes.

Representative western blots of primary hepatocytes pretreated with or without indicated concentrations of PFD followed by GalN/TNF treatment for indicated times. cIAP-1: cellular inhibitor of apoptosis-1, JNK: c-Jun N-terminal kinase.

Supplementary Fig. S5. PFD inhibits Fas-mediated caspase-8 activation, but does not inhibit palmitate-induced caspase-8 and -3 activation in primary hepatocytes.

(a) Representative western blots and (b) quantification of FAS, cleaved caspase-8 and -3 expression of primary hepatocytes pretreated with or without indicated concentrations of PFD followed by treatment of Jo-2 antibody for 6 h. (c) Representative western blots of cleaved caspase-8 and -3 expression of primary hepatocytes pretreated with or without indicated concentrations of PFD followed by treatment of palmitate for 24 h. * $p < 0.05$, ** $p < 0.01$. $n = 3$.

Supplementary Fig. S6. PFD does not affect induction of genes related to fibrosis and inflammation in HSC and macrophage cell lines.

mRNA expression levels in (a) LX-2 and (b) RAW264.7 cells pretreated with or without indicated concentrations of PFD followed by treatment of TGF-β or LPS, respectively. * $p < 0.05$, ** $p < 0.01$. $n = 4$.

(a)

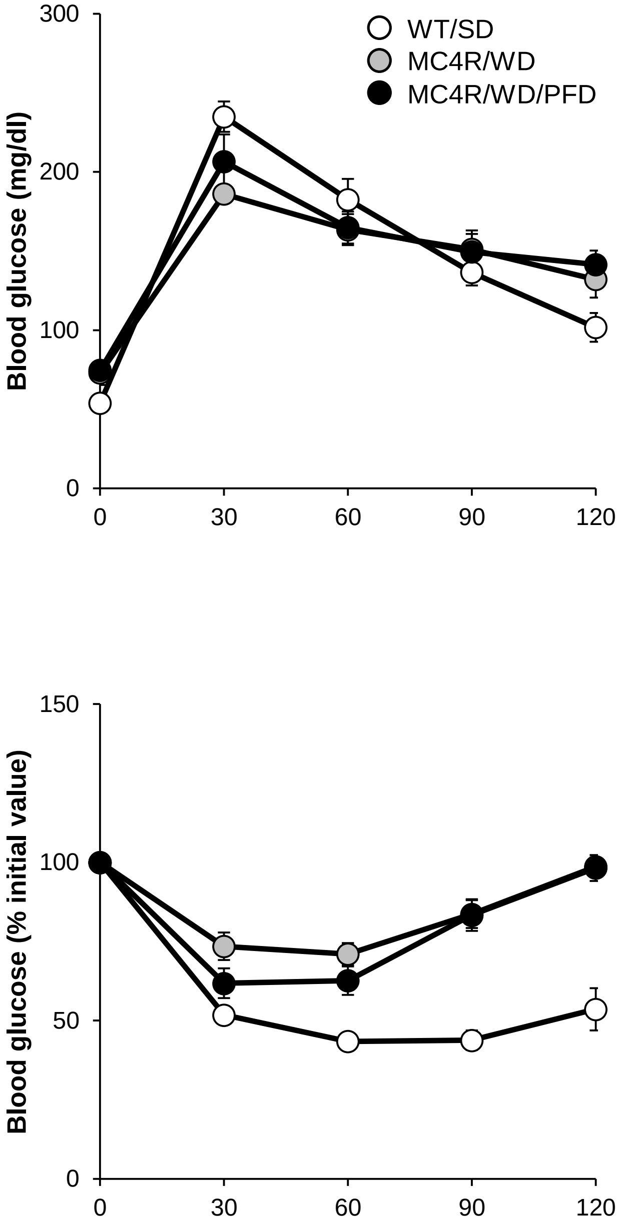

Supplementary Fig. S1

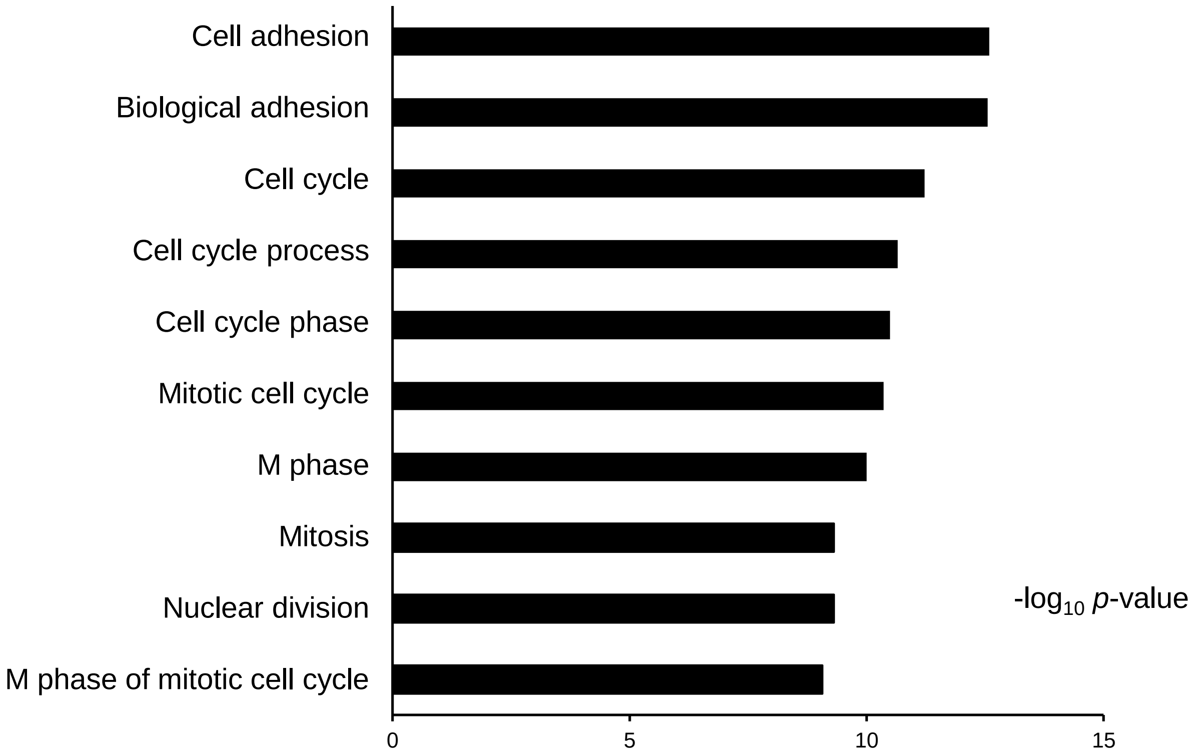

Supplementary Fig. S2

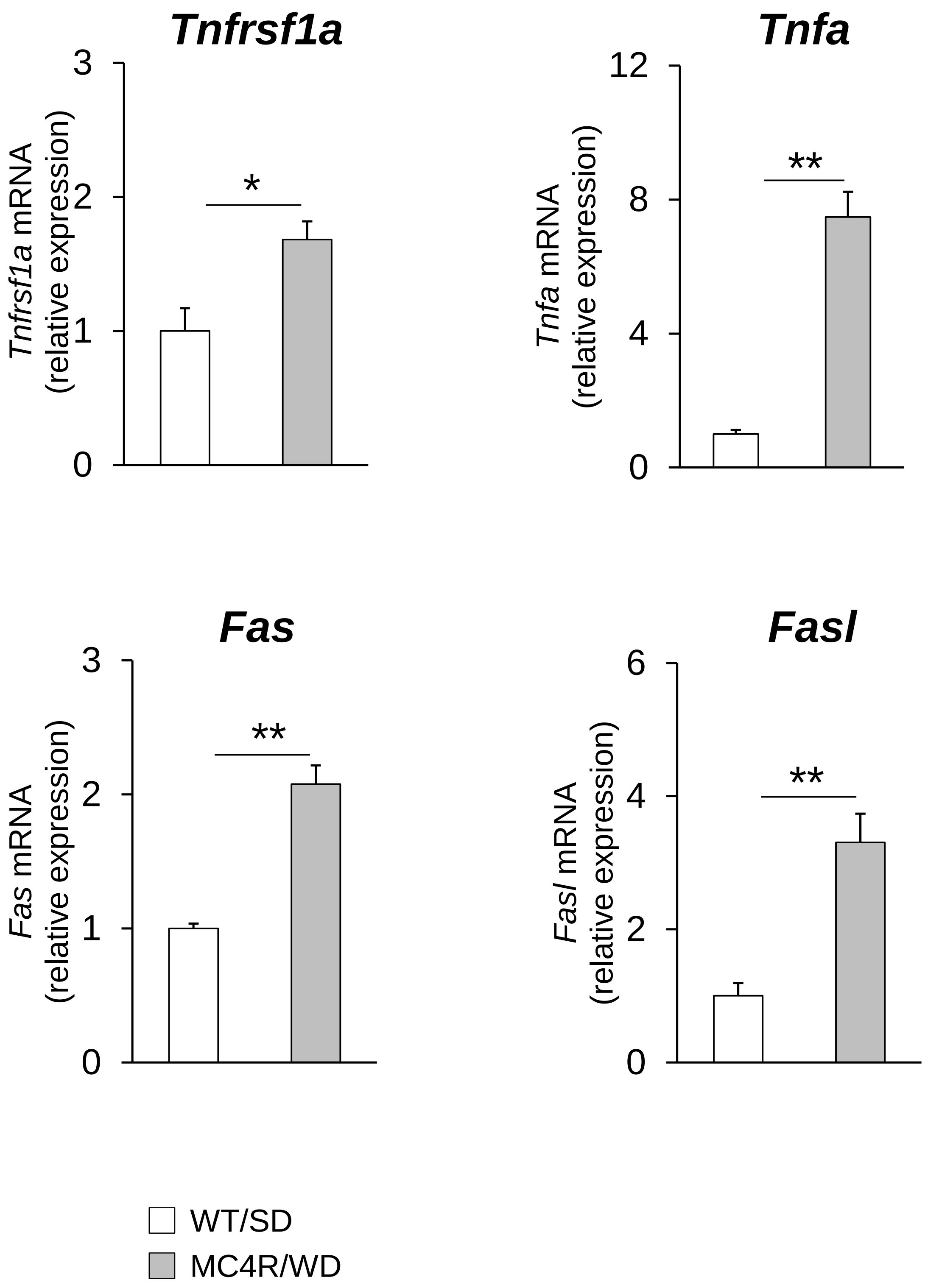

Supplementary Fig. S3

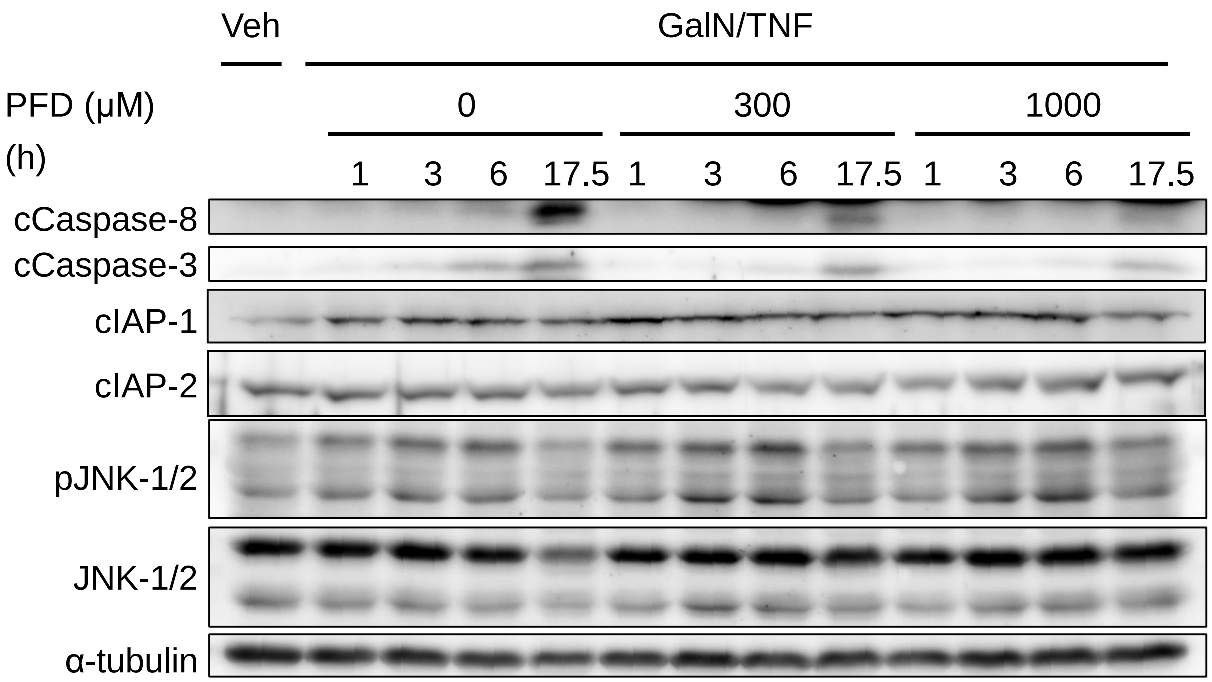

Supplementary Fig. S4

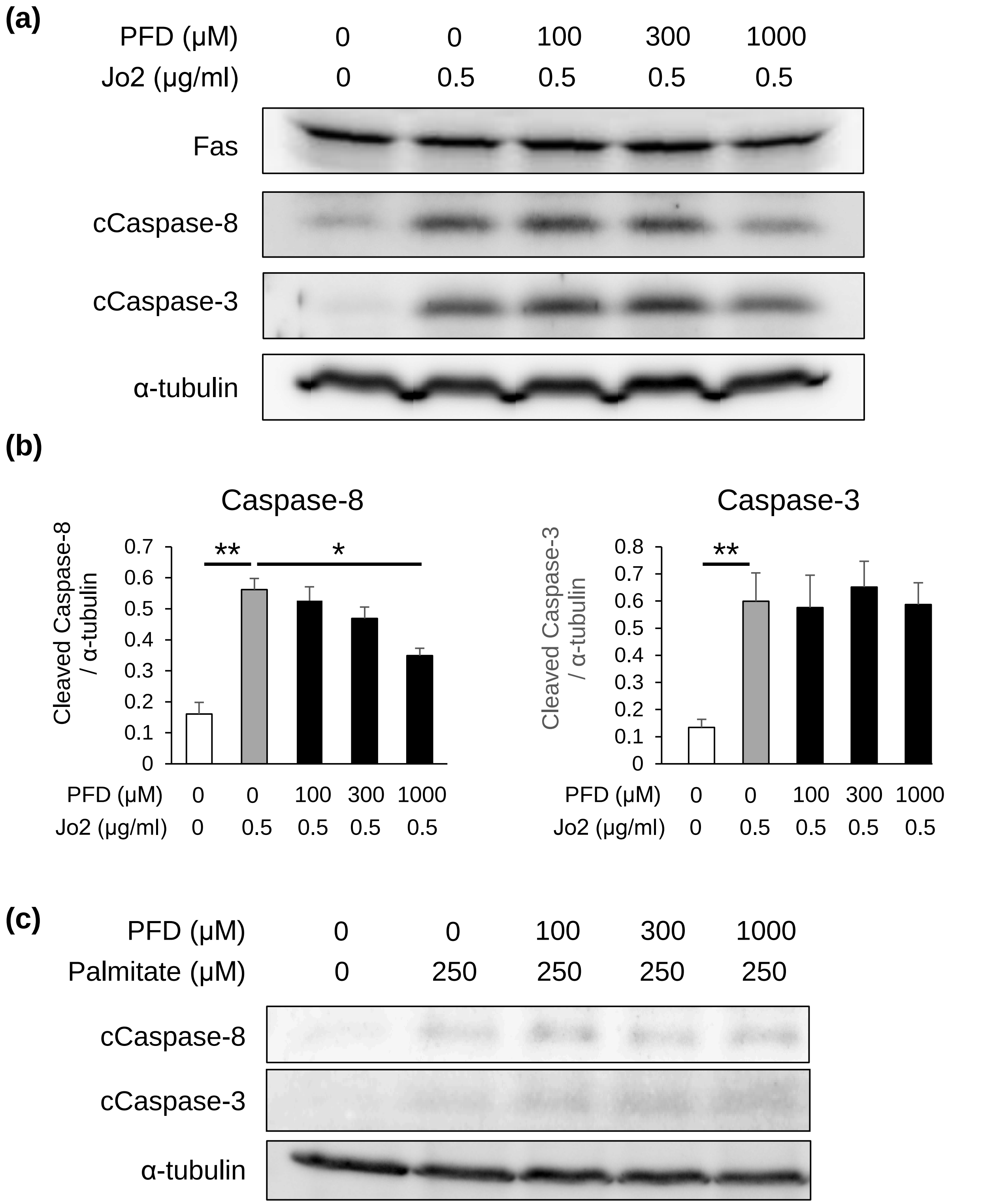

Supplementary Fig. S5

(a)

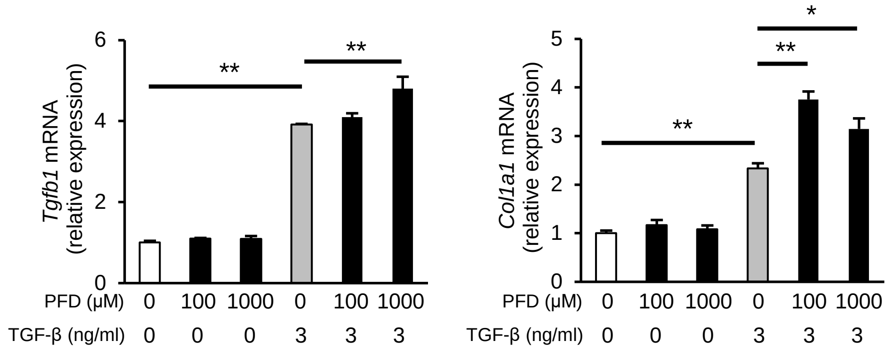

(b)

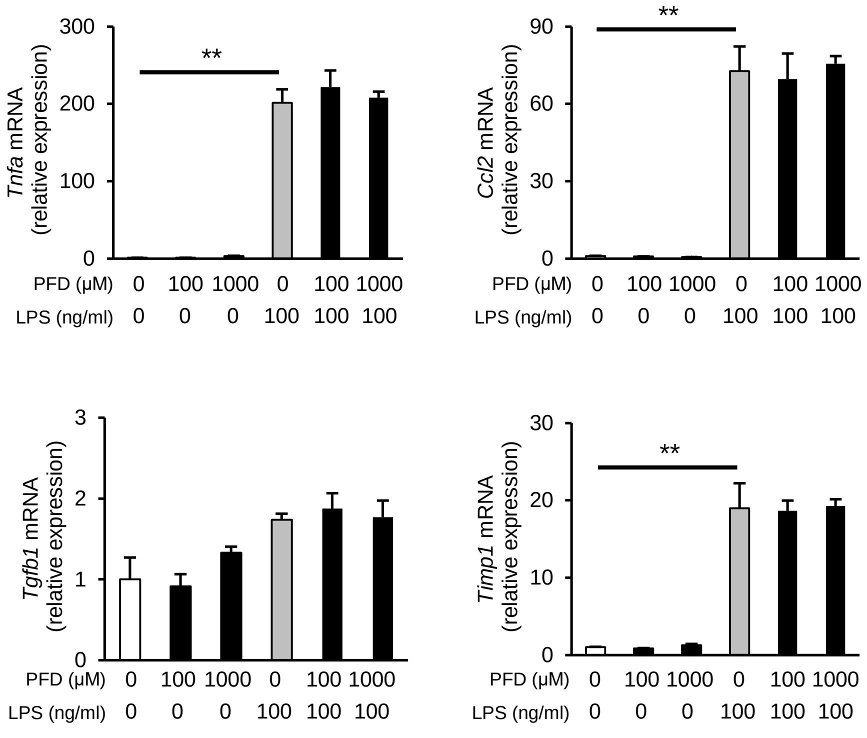

Supplementary Fig. S6
